# The Progression of Prion Diseases Is Not Altered in the Absence of TMEM106B

**DOI:** 10.1523/ENEURO.0043-26.2026

**Published:** 2026-10-01

**Authors:** Francisca Bravo-Risi, Gabriela Alburquerque Orta, Peter J. Kim, Hannah Wylie-Young, Tucker H. Stimming, Paulina Soto, Lisbell Estrada, Joanna L. Jankowsky, Rodrigo Morales

**Affiliations:** ^1^Department of Neurology, The University of Texas Health Science Center at Houston, Houston, Texas 77030; ^2^Doctorado en Ciencias con Mención en Materiales Funcionales, Universidad Bernardo O’Higgins, Santiago 8370993, Chile; ^3^Department of Neuroscience, Baylor College of Medicine, Houston, Texas 77030; ^4^Facultad de Ciencias de la Salud, Escuela de Fonoaudiología, Universidad Bernardo O’Higgins, Santiago 8370993, Chile; ^5^Centro Integrativo de Biologia y Quimica Aplicada, Universidad Bernardo O’Higgins, Santiago 8370993, Chile

**Keywords:** prions, TMEM106B

## Abstract

Transmembrane protein 106B (TMEM106B) is a type II transmembrane glycoprotein localized in late endosomes and lysosomes. This protein has been attributed to relevant roles in disease risk and progression of multiple neurodegenerative disorders. In experimental models, TMEM106B has been linked with the prion-like spread of misfolded tau proteins responsible for Alzheimer's disease and other tauopathies. The role of this protein in the spread of other misfolded proteins, like prions, has not been explored. Here, we tested whether the removal of TMEM106B altered the progression of prion diseases using animal mouse models (male and female). This was carefully tested using different prion dosages, strains, and routes of administration. Our results demonstrate that the absence of TMEM106B does not alter the progression of prion diseases in any of the tested conditions. This data provides supportive evidence on the specificity of TMEM106B in neurodegenerative conditions.

## Significance Statement

TMEM106B has been suggested to play a relevant role in the pathology of tau proteins and the progression of tauopathies and other protein misfolding disorders. Here, we tested whether the lack of TMEM106B affected the development of prion diseases. This was explored using different prion strains inoculated by two routes of administration. Our results demonstrate that the absence of TMEM106B does not seem to modulate the development of prion diseases. This data strongly suggests that TMEM106B contribute to the misfolding of specific proteins and does not play a general role in the context of neurodegeneration.

## Introduction

Transmembrane protein 106B (TMEM106B) is a type II transmembrane glycoprotein localized in late endosomes and lysosomes (Chen-Plotkin et al., 2012; Lang et al., 2012), where it influences lysosomal size, pH, and movement (Stagi et al., 2014; Feng et al., 2021; Zhu et al., 2024; Takahashi and Strittmatter, 2025). Beyond these straightforward effects in cultured cells, mounting evidence suggests that TMEM106B dysregulation can influence disease risk and progression spanning multiple neurodegenerative disorders. Genome-wide association studies first identified TMEM106B as a risk modifier for frontotemporal lobar degeneration (Van Deerlin et al., 2010; Van Der Zee et al., 2011); subsequent studies have extended this association to Alzheimer's disease (AD), hippocampal sclerosis of aging, and limbic predominant age-related TDP-43 encephalopathy (Lu et al., 2014; Nelson et al., 2014, 2019; Milind et al., 2020). Additional reports have linked TMEM106B to cognitive trajectory in Parkinson's disease and ALS (Vass et al., 2011; Tropea et al., 2019). Current studies in animal models suggest that TMEM106B may be protective against neurodegeneration where complete loss of this protein exacerbates disease phenotypes. We and others recently communicated that the lack of TMEM106B in a model of tau pathology accelerated neurodegenerative phenotypes, with worsening cognitive impairment, increased tau pathology, greater brain atrophy, and enhanced neuroinflammation (Edwards et al., 2024; Feng et al., 2024). Similar exacerbation was reported for the GRN null model of frontotemporal dementia upon TMEM106B deletion (Feng et al., 2020a; Werner et al., 2020; Zhou et al., 2020). Both tau overexpression and progranulin deletion cause intraneuronal pathology, suggesting that TMEM106B can influence proteinopathies originating inside the cell. Yet many of the diseases modified by TMEM106B also involve extracellular processes including cell-to-cell transfer, seeding, or plaque formation (Peng et al., 2020). Although TMEM106B has not yet been studied in the context of prion diseases, this group of disorders provides an ideal platform for examining disease processes dependent on extracellular transmission.

Prion diseases are transmissible protein misfolding disorders (PMDs) affecting humans and animals. The replication of the prion infectious agent (PrP^Sc^) is an autocatalytic process where PrP^Sc^ templates its aberrant conformation to the cellular prion protein (PrP^C^; Prusiner, 1982). PrP^C^ has been associated to sphingolipid-sterol rafts at the cell membrane. Specifically, a sphingolipid-binding domain was identified in the C-terminal region of PrP^C^ that interacts with galactosylceramide (GalCer; Mahfoud et al., 2002), a glycosphingolipid found in lipid rafts. Interestingly, TMEM106B participates in myelin lipid metabolism by increasing the lysosomal enzymatic activity of galactosylceramidase that hydrolyzes GalCer. Importantly, GalCer and sulfatide, two of the major glycosphingolipids in myelin, are decreased in *Tmem106b^(−/−)^* (KO) mice (Takahashi et al., 2024). The decrease in galactosylceramide may influence the sphingolipid-binding domain where PrP^C^ associates with. Moreover, prion conversion has been suggested to occur within the endocytic recycling and the endosomal-lysosomal pathways (Yim et al., 2015) where TMEM106B is located. These antecedents suggest potential overlap between TMEM106B and prion conversion.

To understand the role of TMEM106B in protein misfolding progression and investigate the potential link between this protein and prions, we aimed to systematically study the impact of TMEM106B deficiency in the context of prion diseases.

## Material and Methods

### Animal models and bioassays

Male and female wild-type (WT) and *Tmem106b ^(−/−)^* mice were used in this study (Feng et al., 2020b). Mice were generated and maintained on a C57BL/6J background. The mice were transferred from Baylor College of Medicine to UTHealth-Houston approved facilities where they were housed, treated, and humanely killed. Housing conditions are detailed in Soto et al. (2024). Mice of 173 ± 1 d old (means ± SD) were submitted to a single stereotaxic injection in the hippocampus (right hemisphere). The stereotaxic coordinates used were −2.0, −2.0, and −2.0 mm anteroposterior, mediolateral, and dorsoventral, respectively. The coordinates were selected based on a mouse brain atlas (Paxinos and Franklin, 2001). The mice were intracranially (i.c.) challenged with 1 and 0.1% (w/v) brain homogenate (BH), equivalent to 100 and 10 ng of brain tissue containing RML prion (mouse-adapted scrapie) and 1% (w/v) BH of 301C (BSE-derived prion adapted in mouse) infected brains. The intracranial inoculations were performed as described in (Soto et al., 2024)*.* To perform the intracranial inoculations, a Hamilton 701N microliter syringe was used (Hamilton Company, catalog #80366).

The intraperitoneal (i.p.) inoculations were performed using a single dose of 100 µl of 0.1% (w/v) BH containing RML prions, equivalent to 100 ng of brain tissue. The mice were manually restrained to expose the abdomen, and a needle (1 ml syringe with 26-gauge × 3/8 inch needle, Fisher Scientific, catalog #50-209-2978) was inserted in the lower left abdominal quadrant peritoneal cavity (lateral to middle), and the inoculum was administered. The intraperitoneal inoculations were performed by two researchers.

The clinical signs were evaluated weekly, and the animals were humanely killed once they reached an advanced stage of disease. Killing was conducted by CO_2_ inhalation and death was confirmed by decapitation, as described (Soto et al., 2024).

### Brain collection and preparation

The brains from these WT and *Tmem106b^−/−^* mice were collected and frozen at −80°C until use. The brains were thawed on ice, and a 20% (w/v) BH was prepared by homogenizing the brains in a fresh solution of Cytiva HyClone PBS (Global Life Sciences Solutions, catalog #SH30256.02) containing a protease inhibitor cocktail with EDTA (MilliporeSigma, catalog #11697498001). Then, the samples were diluted at 10% (w/v) BH using the same solution. Both the 20 and 10% (w/v) BH solutions were stored at −20°C until use.

### Proteinase K treatment

The 10% (w/v) BH were thawed on ice and a dilution to 5% (w/v) BH was made. Twenty microliters of these 5% (w/v) BH were mixed with proteinase K (PK) from *Tritirachium album* (MilliporeSigma, catalog #P2308) at a final concentration of 100 µg/ml. The reaction was incubated in a ThermoMixer R (Eppendorf, catalog #022670107) at 37°C for 90 min with shaking at 450 rpm. The digestion reaction was stopped by adding 4× NuPAGE LDS Sample Buffer (Fisher Scientific, catalog #NP0007) to a final concentration of 1× and incubating it at 95°C for 10 min.

### PK-resistance PrP screening

The digested samples were loaded onto 12% NuPAGE gels (Life Technologies, catalog #NP0342BOX). One well of the gel was used to load the EZ-Run molecular weight ladder (Fisher Scientific, catalog #BP36031). The electrophoretic conditions were as follows: (1) a first run at 80 V for 20 min and then (2) a second run at 140 V for 100 min. Next, the proteins were transferred using the Trans-Blot Turbo Transfer System (Bio-Rad Laboratories, catalog #1704150) at 2.5 A, 20 V for 7 min. The sandwich was assembled on the tray by using Midi filters (Bio-Rad Laboratories, catalog #12023956), and a nitrocellulose membrane (Global Life Sciences Solutions USA, catalog #10600003). During assembly, membrane and gels were soaked in transfer buffer (Bio-Rad Laboratories, catalog #10026938) prepared according to the manufacturer's instructions. Then, the membranes were blocked in a 10% (w/v) nonfat milk solution for 15 min. PK-resistant PrP was immunolabeled using the purified anti-CD230 (prion) monoclonal antibody clone 6D11 (BioLegend, catalog #808003, RRID: AB_2564737) at a dilution of 1:5,000. The membrane was incubated overnight at 4°C. After rinsing the primary antibody, a secondary antibody [anti-mouse IgG (whole molecule)-peroxidase produce in sheep (Sigma-Aldrich, catalog #A5609)] was applied to membranes at a dilution of 1:3,000 for 60 min at room temperature (RT). Between and after antibody incubations, the membranes were rinsed with washing buffer [PBS containing 0.05% (v/v) Tween-20]. The same buffer was used to prepare the blocking and antibody solutions. Membranes were developed using the Amersham ECL Select Western Blotting Detection Reagent (Global Life Sciences Solutions USA, catalog #RPN2235) prepared according the manufacturer's instructions. Western blot screening was performed by two researchers. The images were acquired using the ChemiDoc XRS image analysis system. The images were analyzed using Image Lab (Bio-Rad Laboratories, version 6.0.1) and Fiji software (ImageJ, version 1.54p; Schindelin et al., 2012) for Microsoft Windows.

### Strain differences

For strain characterization, a set of brain samples per experimental condition was analyzed. These same brain samples were used for further biochemical analyses. Briefly, 10% (w/v) BH were thawed and subjected to PK digestion as described above. Then, the samples were diluted in PBS to a final concentration of 2.5% (w/v). To terminate PK enzymatic activity, each sample was mixed with 4× NuPAGE LDS Sample Buffer and treated under the conditions described previously. In parallel, undigested samples (not subjected to PK digestion) were prepared by adding an equivalent volume of PBS instead of PK, followed by dilution to 2.5% (w/v). LDS was added identically and treated under the same conditions to the digested samples. All samples were stored at −20°C until used. For Western blotting, 20 µl of sample was loaded into 12% NuPAGE gels, alternating between undigested (PK−) and digested (PK+) samples. The fractioning and WB were conducted as described previously. Membranes were immunolabeled using purified mouse anti-prion protein monoclonal antibody, clone 8H4 (Sigma-Aldrich, catalog #P0110, RRID: AB_1079676). This antibody was diluted in a 1:5,000 ratio in PBST.

### Quantification of PrP^Sc^ levels

To evaluate PrP^Sc^ levels, a semiquantitative analysis was conducted. Briefly, a set of three brain samples from mice inoculated with either 1% (w/v) RML- and 301C-infected BHs were analyzed. Brains from healthy WT and *Tmem106b ^(−/−)^* mice were included as negative controls. Eighty microliters of 10% (w/v) BH were treated with PK as described above, reaching a final concentration of 8% (w/v) during the digestion. Following digestion, the reactions were diluted in PBS to a 5% (w/v) BH and used to prepare twofold serial dilutions ranging from 2.500 to 0.156% (w/v; equivalent to a range of 625.0–39.0 mg of brain tissue). Finally, an equal volume of 4× LDS buffer was added to each tube and treated under the heating conditions described above. The entire volume of each reaction was loaded into 12% NuPAGE gels. The fractioning and Western blotting were conducted as described previously. Membranes were immunolabeled using a purified mouse anti-prion protein monoclonal antibody, clone 8H4. This antibody was diluted in a 1:5,000 ratio in PBST.

### Evaluation of PrP^C^ expression levels

To evaluate the PrP^C^ expression levels in the brains from healthy WT and *Tmem106b^(−/−)^* mice, the total protein concentration in 10% (w/v) BH was first determined. Samples were diluted 1:50 in PBS and total protein quantified using a Pierce BCA protein Assay Kit (Thermo Fischer Scientific, catalog #23227), according to manufacturer's instructions. The analyses included three technical replicated per biological sample. Thereafter, deglycosylation with PNGase F (New England Biolabs, catalog #P0704L) was conducted under denaturing conditions. Briefly, 45 µl of 10% (w/v) BH was mixed with 5 µl of 10× Glycoprotein Denaturing Buffer. Denaturation was achieved by incubating the mix at 100°C for 10 min. Following denaturation, tubes were placed on ice and centrifuged. Next, 7 µl of 10× GlycoBuffer 2, 7 µl of 10% NP-40, and 6 µl of HyPure Molecular Biology Grade Water (HyClone Laboratories, catalog #SH30538.02) were added to the reaction. One microliter of PNGase F was then added to each tube, and the mix was incubated at 37°C for 1 h. To load 50 µg of total protein per well, the reactions were adjusted by adding PBS and LDS at a final concentration of 1×. Subsequently, the samples were incubated at 95°C for 10 min and stored at −20°C until use. Electrophoresis was performed under the following conditions: an initial run at 80 V for 20 min, followed by a second run at 120 V for 1 h and 25 min. Proteins were transferred to a nitrocellulose membrane as described above. Following transfer, total proteins were stained with filtered Ponceau S (Research Products International, catalog #P56200-500) for 15 min at room temperature. Membranes were rinsed with dH_2_O until background-to-signal was optimal, and images were immediately acquired. The Ponceau S stain was then completely removed using dH_2_O. Next, for PrP detection, membranes were blocked, immunolabeled, and developed as described above, with the exception of the primary used. Here, the mAb 8H4 was used at 1:5,000 dilution in PBST and incubated for 1 h at room temperature.

### Semiquantitative PrP glycoform analysis

From the Western blots obtained in the “Strain differences” analyses, the relative optical density (ROD) area of the distinct post-translational PrP^Sc^ glycoforms (di-, mono-, and un- glycosylated forms) was quantified. The total PrP^Sc^ signal was calculated as the sum of the individual glycoform areas. The percentage of each specific glycoform was determined using the following formula:

$$\mathrm{Glycoform}\;(\text{\%})=\frac{\mathrm{Area}\;\mathrm{of}\;\mathrm{individual}\;\mathrm{glycoform}\;(\mathrm{ROD})}{\mathrm{Total}\;\mathrm{Area}\;(\mathrm{ROD})}\times 100.$$

Next, for statistical analysis, the diglycosylated band was selected to be compared between experimental conditions.

### Quantification of PrP^Sc^ levels analysis

From the Western blots obtained in the “Quantification of PrP^Sc^ levels” analysis, the ROD area of PrP^Sc^ signal was quantified using the signal depicted at concentration of 0.625% (w/v; equivalent to 156.25 mg of brain tissue), per animal. The relative PrP^Sc^ burden among the experimental groups was calculated with the equation below, and the resulting means were statistically compared with evaluate differences in relative PrP^Sc^ levels between groups:

$$\mathrm{Relative}\;\mathrm{PrPSc}\;\mathrm{Burden}=\frac{\mathrm{Relative}\;\mathrm{Area}\;\;\mathrm{of}\;\mathrm{PrPSc}\;\;\mathrm{burden}\;(\mathrm{ROD})}{\mathrm{Mass}\;\;\mathrm{of}\;\mathrm{brain}\;\mathrm{tissue}\;(\mathrm{mg})}.$$

### Evaluation of PrP^C^ expression levels analysis

From the Western blots obtained in the “Evaluation of PrP^C^ expression levels” analysis, the ROD areas of total protein and PrP^C^ signals were quantified. For WT and *Tmem106b^−/−^* mice, three and four biological replicates were analyzed, respectively, with three technical replicates per biological replicate. Membranes stained with Ponceau S were utilized to determine the total protein-stained area. The PrP^C^ area was calculated by conducting densitometric analysis of full-length PrP. The relative expression ratio of PrP^C^ was calculated with the following formula:

$$\mathrm{Relative}\;\mathrm{Expresion}\;\mathrm{Ratio}\;\mathrm{of}\;\mathrm{PrPC}=\frac{\;\mathrm{Relative}\;\mathrm{Area}\;\mathrm{of}\;\mathrm{PrPC}\;\mathrm{burden}\;(\mathrm{ROD})}{\mathrm{Relative}\;\mathrm{Area}\;\mathrm{of}\;\mathrm{Total}\;\mathrm{Protein}\;(\mathrm{ROD})}.$$

### Densitometric and statistical analyses

Densitometric analyses for each experimental group were conducted on the same membrane using 8 bit images. To prevent background signal interference, background subtraction was applied using a rolling ball radius of 85.0 pixels with the light background option using Fiji software (ImageJ, version 1.54p; Schindelin et al., 2012). Statistical analyses and graphing were generated using GraphPad Prism version 10.1.2 for Microsoft Windows (GraphPad Software, www.graphpad.com) on a Microsoft Windows 11 operating system. The Mantel–Cox test was applied for survival curve analysis, the two-way ANOVA with the two-way Tukey's multiple-comparisons test was applied to the di-glycosylation analysis, the two-way ANOVA Šídák's multiple-comparisons test was applied for the relative PrP^Sc^ burden, and the nested *t* test was applied for the PrP^C^ expression level analysis.

## Results

To investigate whether constitutive loss of TMEM106B alters disease progression caused by prion inoculation, we assessed disease onset (incubation period) and terminal prion biochemistry (PrP^C^ levels, protease-resistant PrP, and glycosylation forms) in WT and KO mice across three experimental variables: (1) inoculum concentration; (2) prion strain, RML (mouse-adapted scrapie) or 301C (BSE-derived prions adapted in mice); and (3) route of inoculation—intracranial (i.c.) or intraperitoneal (i.p.). Before proceeding with these experiments, we analyzed whether the absence of TMEM106B altered the expression of the cellular prion protein (PrP^C^). This might have relevant consequences in prion infectivity, as incubation periods are indirectly proportional to the expression of the prion protein (Fischer et al., 1996). Our studies demonstrate no significant differences in bulk PrP^C^ levels in the brain of mice expressing or lacking TMEM106B (Fig. 1), indicating that TMEM106B deletion does not measurably alter total PrP^C^ expression under these conditions.

**Figure 1.**
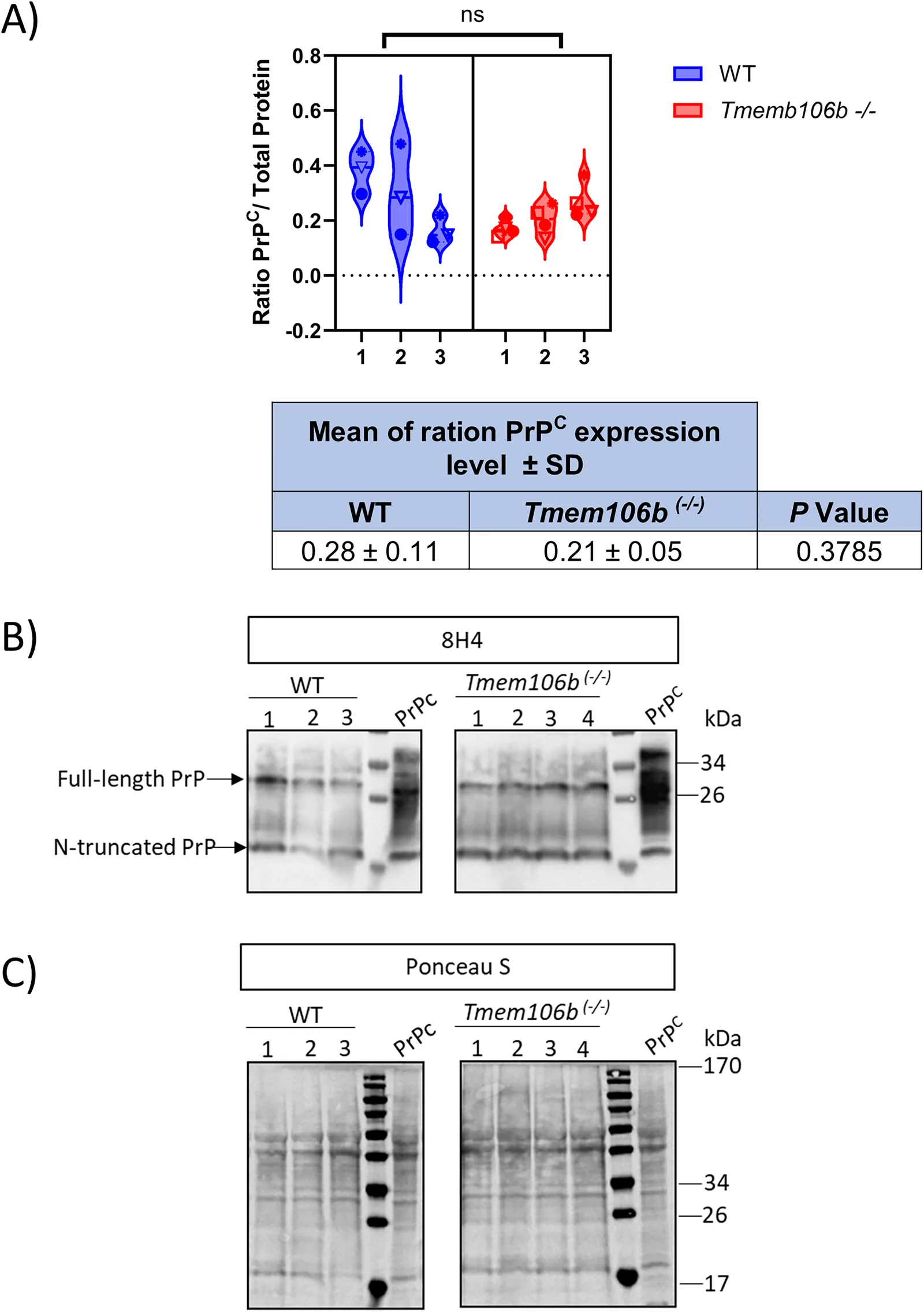
PrP^C^ levels in *Tmem106b*^(^*^−/−^*^)^ and WT mice. ***A***, Densitometric semi quantification analysis of the relative PrP^C^ levels in *Tmem106b*^(*−/−*)^ (red) and WT (blue). Statistical analysis was performed using a two-tailed nested *t* test (*p* = 0.3785, ns: no significance, *p* value >0.05). The bottom table summarizes the mean ± SD of PrP^C^ to total protein ratio. Numbers in the *x*-axis represent different animals (biological replicates). Dots within each column depict technical replicates. ***B***, Representative Western blot showing PrP^C^ levels in brain homogenates from healthy, noninoculated WT and *Tmem106b^(−/−)^* mice. ***C***, Representative Ponceau S stain showing total protein load for the panel shown in ***B***. All samples were deglycosylated with PGNase F (500 Units) prior analysis. After enzymatic removal of PrP N-link glycans resolves two bands in “***B***”: full-length PrP and the N-terminally truncated PrP. Labels at the top of the membranes indicate the mouse genotype, detection method, and biological replicates. Numbers to the right depict molecular weight in KDa. PrP^C^, WT mouse brain extracts without protease treatment used as electrophoretic mobility and antibody reactivity control. Both WT and *Tmem106b*^(*−/−*)^ were analyzed with *n* = 3 and *n* = 4 biological replicates, respectively, analyzed across *n* = 3 technical replicates.

To determine whether TMEM106B influences the sensitivity to prion dose, we introduced two different concentrations of RML prions into the hippocampus by stereotaxic injection (1 and 0.1% w/v brain extract)*.* As expected, lower inoculate concentration delayed incubation periods (Fig. 2*A*,*B*; Table 1). However, within each dose, we found no significant differences in incubation periods between WT and KO mice.

**Figure 2.**
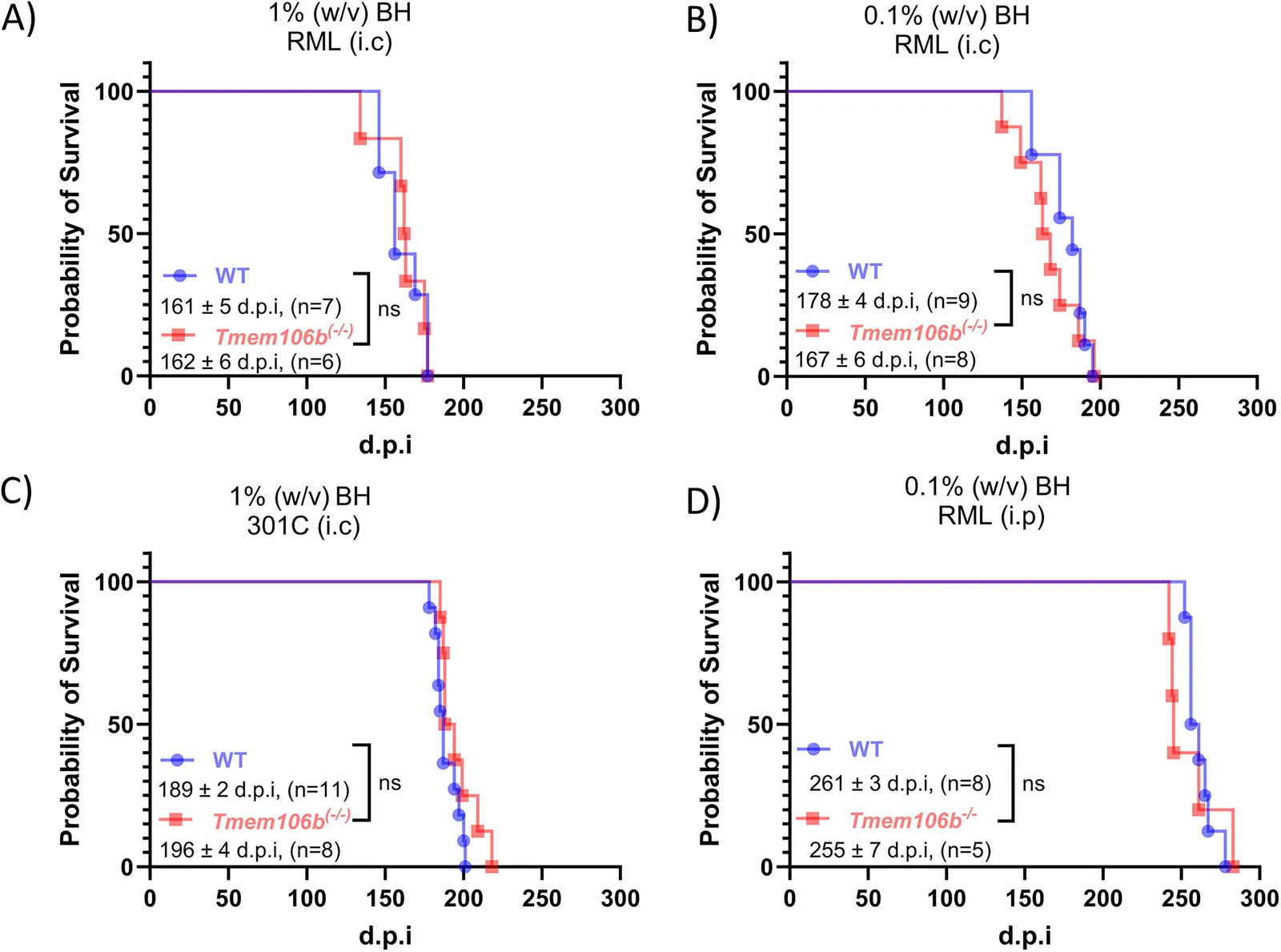
Incubation periods of prion infected *Tmem106b*^(*−/−*)^ and WT mice challenged by different prion strains and routes of administration. Survival curves of wild-type (WT, blue) and TMEM106B knock-out (*Tmem106b^(−/−)^*, red) mice under different experimental paradigms: ***A***, Mice intracerebrally (i.c.) inoculated with 1% (w/v) brain extracts containing RML prions. ***B***, Mice intracranially inoculated with 0.1% (w/v) brain extracts containing RML prions. ***C***, Mice intracranially inoculated with 1% (w/v) brain extracts containing 301C prions. ***D***, Mice intraperitoneally (i.p.) inoculated with 0.1% (w/v) brain extracts containing RML prions. Incubation periods are expressed as mean ± SEM in days post-inoculation (dpi). *n*, number of mice inoculated in each group. Statistical differences were determined using the Mantel–Cox test. ns, *p* value >0.05.

**Table 1.** Transmission characteristics of RML and 301C prions

Group	Inoculum (dilution BH)	Inoculation route	Incubation period^a^	Attack rate^b^ (%)	PK-resistant PrP prevalence^c^ (%)	*p* value
WT	RML (10^−2^)	i.c	161 ± 5	7/7 (100%)	7/7 (100%)	0.9435
*Tmem106b ^(−/−)^*			162 ± 6	6/6 (100%)	6/6 (100%)	
WT	RML (10^−3^)	i.c	178 ± 4	9/9 (100%)	9/9 (100%)	0.4745
*Tmem106b ^(−/−)^*			167 ± 6	8/8 (100%)	9/9 (100%)	
WT	301C (10^−2^)	i.c	189 ± 2	11/11 (100%)	11/11 (100%)	0.1324
*Tmem106b ^(−/−)^*			196 ± 4	8/8 (100%)	7/7^d^ (100%)	
WT	RML (10^−3^)	i.p	261 ± 3	8/8 (100%)	8/8 (100%)	0.7922
*Tmem106b ^(−/−)^*			255 ± 7	5/5 (100%)	5/5 (100%)	

This table details depicts group survival data illustrated in Figure 2.

a Mean ± SEM in days.

b Number of mice with clinical prion disease/number of mice inoculated.

c Number of mice with PrP^Sc^ signal by WB/number of mice inoculated.

d Brain sample from animal 47-1 not analyzed.

Next, we asked whether TMEM106B loss modulates disease progression in a prion strain-specific manner. Prion strains are known to target different brain regions and cell types inducing different toxic responses in neurons. We challenged WT and KO mice with the well-characterized 301C prion strain [1% (w/v) brain extract], a BSE-derived prion adapted in mice. Both genotypes displayed comparable incubation periods with no significant differences in survival time (Fig. 2*C*, Table 1). This data suggests that TMEM106B deletion does not alter terminal incubation time when challenged with either RML or 301C prion strains.

Until now we focused on intracerebral injections to expose prions directly to the brain. However, prions can also spread to the brain from peripheral exposure. After peripheral inoculation, prions replicate in secondary lymphoid organs (e.g., spleen, and lymph nodes) and retrogradely spread in the peripheral nervous system before invading the brain (Koshy et al., 2022). Prion dissemination varies with the route of exposure, producing distinct peripheral tropisms along anatomical pathways and relying on specific cell types, including follicular dendritic cells, M cells, astrocytes, and neurons (Vilette et al., 2018). In contrast, intracerebral administration places infectious particles directly into the brain. In both settings, prion uptake and spread are thought to involve the endo/lysosomal system (Kovács et al., 2007; Shim et al., 2016; Fehlinger et al., 2017). Given TMEM106B's role in endo/lysosomal trafficking (Feng et al., 2020a), and in tau misfolding in vivo (Edwards et al., 2024; Feng et al., 2024), we asked whether it might also influence prion spread and neuroinvasion. We tested this by monitoring incubation periods in WT and KO mice after intraperitoneal administration of RML prions. As expected, peripheral prion exposure prolonged incubation periods compared with intracranial inoculations (Fig. 2*B*,*D*; Table 1). However, survival times showed no statistically significant differences between genotypes regardless of exposure route (Fig. 2*B*,*D*).

We further analyzed whether the absence of TMEM106B altered the strain features of RML and 301C prions. RML prions are characterized by an enrichment of the monoglycosylated band, and an electrophoretic mobility of ∼21 kDa for the unglycosylated band. On the contrary, 301C preferentially recruits diglycosylated prion isoforms, while displaying an unglycosylated band at ∼19 kDa (Castilla et al., 2008). Analyses on electrophoretic mobilities and glycosylation profiles of PK-resistant prions did not show changes of prion strain patterns for the prions replicated in WT and KO mice (Fig. 3*A*,*B*). Semiquantitative analysis of the diglycosylated fraction also did not reveal genotype-dependent differences either (Fig. 3*C*). This suggests that TMEM106B does not alter the glycosylation signatures associated with RML or 301C prions.

**Figure 3.**
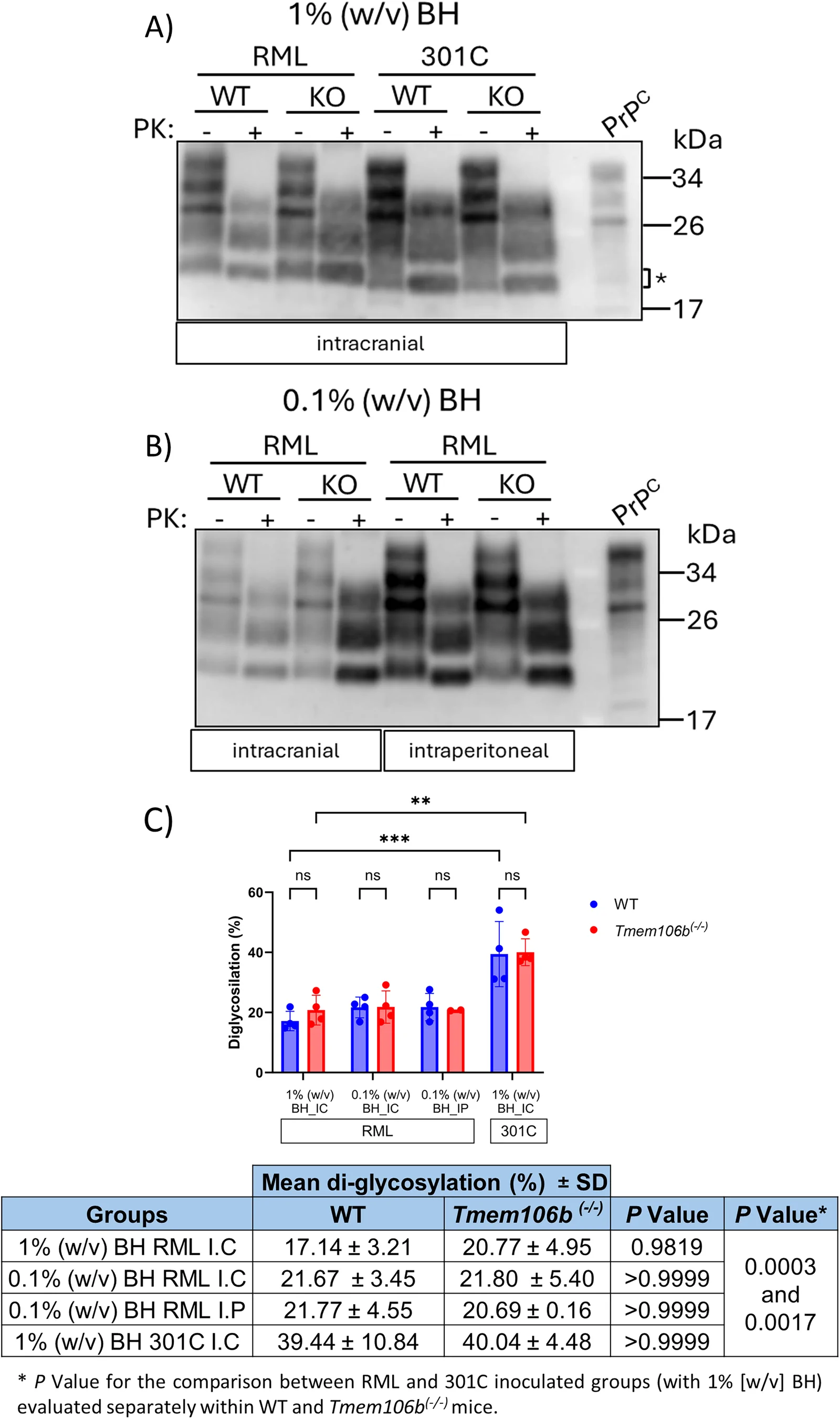
The biochemical properties of RML and 301C prions are maintained in both *Tmem106b*^(^*^−/−^*^)^ and WT mice. ***A***, Representative Western blot of brain homogenates from WT and *Tmem106b^(−/−)^* (KO) mice inoculated intracranially (IC) with 1% (w/v) brain extracts containing either RML or 301C prions. ***B***, Representative Western blot of brain homogenates from WT and *Tmem106b^(−/−)^* mice inoculated either intracranially or intraperitoneally (IP) with 0.1% (w/v) brain extracts containing RML prions. In ***A*** and ***B***, samples were either untreated (−) or treated (+) with PK. Labels at the top of the blot indicate the prion strain, inoculation dose, and PK treatment status. Labels below the blot indicate the route of inoculation. Numbers at the right of the panels depict molecular weight marker (in KDa). The bracket (“]*”) at the right highlights the characteristic ∼2 kDa shift in electrophoretic mobility between the nonglycosylated PrP^Sc^ bands of RML and 301C prion strains (Piro et al., 2009). PrP^C^, WT mouse brain extracts without protease treatment used as electrophoretic mobility and antibody reactivity control. ***C***, Semiquantification of diglycosylated band percentage across treatment groups, comparing between WT (blue) and *Tmem106b^(−/−)^* (red) mice. Statistical significance was evaluated using a two-way ANOVA with the two-way Tukey's multiple-comparisons test. (**p < 0.01, ***p < 0.001, ns, no statistically significant, *p* > 0.05). The summary table display the mean ± SD and *p* values for each comparison. Data represent *n* = 4 biological replicates per condition, except for the *Tmem106b^(−/−)^* mice intraperitoneally inoculated with RML prions (*n* = 2).

Finally, we analyzed if the absence of TMEM106B altered the accumulation of prions in infected mice. We quantified the PrP^Sc^ levels in the brains of terminally ill, WT, and KO animals inoculated with 301C and RML prions. The densitometric analysis of PK-resistant PrP^Sc^ bands showed no statistically significant differences in the prion levels when comparing by mouse background (Fig. 4). This result indicates that, at terminal endpoint, TMEM106B loss does not produce large changes in bulk PrP^Sc^ accumulation for both 301C and RML prions.

**Figure 4.**
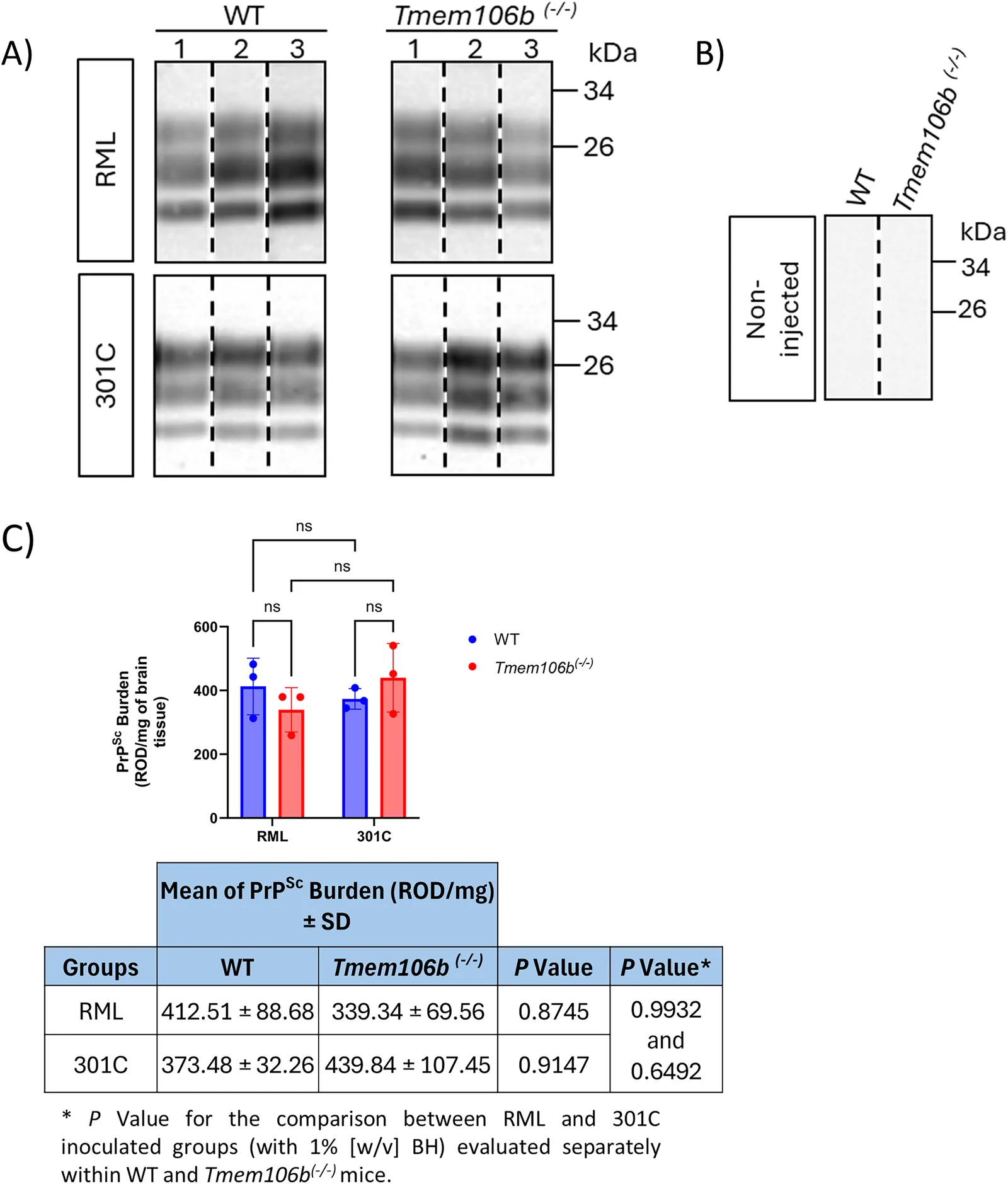
PrP^Sc^ levels in *Tmem106b*^(^*^−/−^*^)^ and WT mice intracerebrally challenged with RML and 301C prions. ***A***, Representative Western blot depicting brain homogenates of WT and *Tmem106b^(−/−)^* mice inoculated intracranially with 1% (w/v) brain extracts containing either RML or 301C prions. ***B***, Representative Western blot depicting brain homogenates from healthy WT and *Tmem106b^(−/−)^* control mice. All brain extracts were digested with PK. Signals correspond to PK-resistant PrP from 0.625% (w/v) BH (equivalent to 156.25 mg of brain tissue). Labels at the top of the blot indicate mouse genotype and biological replicate number. Labels at the left indicate the prion inocula. Molecular weight, in kDa, are shown at the right. Dashed lines indicate cropping locations within the same membrane. ***C***, Quantification of relative PrP^Sc^ levels comparing between WT (blue) and *Tmem106b^(−/−)^* (red) mice across all conditions. Statistical significance was assessed using a two-way ANOVA Šídák's multiple-comparisons test. (ns, not significant, *p* value >0.05). Summary table displays mean ± SD and *p* values. Data represent *n* = 3 biological replicates per group, except noninoculated controls (*n* = 1).

## Discussion

Although TMEM106B has been implicated in diverse protein misfolding diseases such as FTLD, ALS, AD, and tauopathies, our findings suggest that its absence does not detectably alter prion incubation times or terminal PrP^Sc^ biochemistry under the specific dose, strains, and inoculation routes tested here. This is supported by the fact that the absence of TMEM106B did not alter (1) the prion strain glycoform and mobility features associated with RML and 301C strains, nor (2) the accumulation of PrP^Sc^ burden at terminal stages of the disease. Our findings align with a recent study in TMEM106B hypomorphic mice, which reported that deletion had no effect on tau seeding in vivo (Nies et al., 2021). Taken together with prior work in tau and GRN models, our data supports the idea that TMEM106B's influence on protein misfolding may be selective, rather than universal, and may depend on the specific proteinopathy and disease context. This work offers the first evidence that, in contrast to tau- and GRN-related models where TMEM106B exacerbates pathology, TMEM106B loss does not appear to significantly modulate the pathogenesis of transmissible spongiform encephalopathies. Further studies using additional and naturally occurring prion strains, as well as histological analyses, will be important to determine whether more subtle or context-dependent roles for TMEM106B in prion biology exist.

## Synthesis

Reviewing Editor: Laura Volpicelli-Daley, The University of Alabama at Birmingham Decisions are customarily a result of the Reviewing Editor and the peer reviewers coming together and discussing their recommendations until a consensus is reached. When revisions are invited, a fact-based synthesis statement explaining their decision and outlining what is needed to prepare a revision will be listed below. The following reviewer(s) agreed to reveal their identity: Robert Mercer.

Both reviewers felt the manuscript provides advances in the field. TMEM106B is a risk factor for multiple neurodegenerative disorders and their fibrils accumulate in the brains of proteinopathy patients. Thus, understanding if this transmembrane protein is relevant for the prion pathogenesis is important for the neurodegeneration research field. This study would provide definitive data that TMEM106B does not play a relevant role on the progression of prion diseases. This negative data show that, while proteinopathies share many pathogenic mechanisms, neuroscientists should not overgeneralize their discoveries.

Both reviewers thought the following would be important for the scientific record and to solidify the conclusions:

1. Although histopathological analyses is a lot of work it is important to provide. Please assess PrPSc burden in the brains of the mice with histology methods and quantify.

2. The WT and Tmem109b-/- homogenates should be on the same blot, + and - PK, for a proper comparison. This arrangement might reveal some differences in PrP between these samples that cannot be appreciated otherwise. Please also comment on the electrophoretic mobility and banding pattern of the prions in both genotypes.

Other minor comments were suggested:

-To expand beyond the pathologic role of TMEM106B in neurodegenerative disorders, it is known that TMEM106B acts as a genetic modifier for cognitive trajectory in Parkinosn's disease (Tropea, ANN NEUROL, 2019)

-Line 43, 44 - a complementary rationale for studying TMEM106B in prion diseases is that the prion conversion seems to occur in the endocytic recycling pathway, and the endosomal-lysosomal pathway, where TMEM106B is located.

-Line 151- Authors claim that "TMEM106B loss does not significantly modulate the pathogenesis of transmissible spongiform encephalopathies." There are too many prion strains to conclude on all. We suggest to conclude: TMEM106B loss does not SEEM TO significantly modulate the pathogenesis of transmissible spongiform encephalopathies.

## Author Response

Dear Editor, Please find enclosed a point-by-point response to the reviewers' comments regarding our submission titled "The progression of prion diseases is not altered in the absence of TMEM106B".

Next, we provide responses to each of the reviewers' comments. These are detailed, below:

1. Although histopathological analyses is a lot of work it is important to provide. Please assess PrPSc burden in the brains of the mice with histology methods and quantify.

Answer: Measuring the burden (area reactive to misfolded protein aggregates versus total area analyzed) for the diffuse aggregation displayed by 301C and RML prions is difficult to quantify by histological means (not straightforward compared with plaque-type deposition observed for other prion strains). Along this line, we did not collect brains for histological analyses. However, to address the reviewers' concern, we measured the PrPSc levels in the brain of experimental and control mice. We didn't observe any significant difference in the brains of treated mice. This data is now shown in Figure 3.

2. The WT and Tmem109b-/- homogenates should be on the same blot, + and - PK, for a proper comparison. This arrangement might reveal some differences in PrP between these samples that cannot be appreciated otherwise. Please also comment on the electrophoretic mobility and banding pattern of the prions in both genotypes.

Answer: We followed the reviewers' comments and conducted the analyses as requested. Although we observed the expected changes between RML and 301C prions, we did not note any electrophoretic profile differences between WT and tmem106b(-/-) mice inoculated with the same prion strain. These analyses include electrophoretic mobility and quantification of the diglycosylated band (hallmarks to differentiate between these two prion strains). This data is now shown in Figure 2.

3. Other minor comments were suggested: a. To expand beyond the pathologic role of TMEM106B in neurodegenerative disorders, it is known that TMEM106B acts as a genetic modifier for cognitive trajectory in Parkinosn's disease (Tropea, ANN NEUROL, 2019) Answer: We have added the reference and antecedent, as suggested. b. Line 43, 44 - a complementary rationale for studying TMEM106B in prion diseases is that the prion conversion seems to occur in the endocytic recycling pathway, and the endosomal-lysosomal pathway, where TMEM106B is located.

Answer: We thank the reviewer for pointing this out. We have added this rationale in the introductory section of the manuscript. c. Line 151- Authors claim that "TMEM106B loss does not significantly modulate the pathogenesis of transmissible spongiform encephalopathies." There are too many prion strains to conclude on all. We suggest to conclude: TMEM106B loss does not SEEM TO significantly modulate the pathogenesis of transmissible spongiform encephalopathies.

Answer: We agree with the reviewer comment and have modified the text and our conclusions, accordingly.

In addition to the above-mentioned changes, we added an additional figure showing that the PrP expression levels are not different between wild type and Tmem106b(-/-) mice. This data is listed in Figure 1.

We are really grateful for your consideration and the comments of the reviewers. As a consequence, we added additional text and 3 new figures. We hope that these modifications are appropriate and support the publication of this manuscript in eNeuro.

I look forward to hear from you.

Sincerely, Rodrigo Morales, PhD Max and Rochelle Levit Distinguished Professor in Neurosciences Department of Neurology The University of Texas Health Science Center at Houston
